# Predictors of the use of community mental health services

**DOI:** 10.1186/s12913-026-14578-z

**Published:** 2026-05-18

**Authors:** Lachlan Cameron, Matt Sutton, Rachel Meacock

**Affiliations:** Health Organisation, Policy and Economics, Division of Population Health, Health Services Research and Primary Care, School of Health Sciences, University of Manchester, Manchester, UK

**Keywords:** Community care, Mental health, Health services, Inequalities

## Abstract

**Background:**

Healthcare systems worldwide are struggling to keep up with the demand created by rising rates of mental health problems. There is a push to increase provision of mental health services in community settings to reduce the strain on hospitals and primary care. However, there is currently very limited evidence on predictors of community mental health service use. This limits our ability to direct resources to people and areas with greater needs, and to identify inequalities in access to services.

**Methods:**

We obtained data on use of community mental health services for 22,277 adult respondents in England from three nationally representative survey data sets. We used logistic regression to relate service use to 14 individual and three area characteristics, and explored whether predictors of service use have changed over time. We then discuss whether the findings suggest variations in access or needs.

**Results:**

In addition to poor mental and physical health, we find that being younger, single, and not employed are associated with higher likelihood of community mental health service use. These findings likely indicate greater need for services. We also find that living in a rural area, the North West region, or being of Asian ethnicity are predictors of lower use. We contend that these results highlight potential inequalities in access. Despite rising rates of service use, these patterns of predictors are consistent over time.

**Conclusions:**

These findings can help inform where additional resources for community mental health services are best directed to reduce the strain on services, and improve access and outcomes.

**Clinical Trial Number:**

Not applicable.

## Introduction

Worldwide, healthcare systems are in crisis, with supply of services failing to keep pace with rising demands. Globally, an estimated 13% of people are living with a mental health problem, with the number of people living with a mental health problem having increased by 25% from 2000 to 2019 (mostly due to rising population) [1]. Many people with mental health problems are not receiving adequate treatment [1–3]. Common barriers include lack of funding, lack of developed mental healthcare policies, and stigma [1, 3]. The World Health Organisation (WHO) have recommended systems to up-scale mental health care in community settings as an approach to improving mental healthcare worldwide, through promoting a more “person-centred, recovery-oriented and rights-based” service [4]. Such community mental health (CMH) services can be defined as “comprising the principles and practices needed to promote mental health for a local population by: a) addressing population needs in ways that are accessible and acceptable; b) building on the goals and strengths of people who experience mental illnesses; c) promoting a wide network of supports, services and resources of adequate capacity; and d) emphasizing services that are both evidence-based and recovery-oriented” [5]. It is hoped that these CMH services can shift some care away from hospitals, helping to reduce the strain on healthcare systems and provide more timely and person-centred care to improve individual mental health outcomes [6].

Despite such aims to scale up provision of CMH services, little evidence exists on the characteristics of service users and drivers of demand. To maximise the benefits of CMH services, it is important to understand what individual demographics, health and health needs, and area effects predict use of CMH services. This evidence is needed to direct resource allocation towards populations with greater need for services, and to explore possible inequalities in use which may arise due to access barriers for certain groups. The existing evidence on predictors of CMH service use is weak because studies either (i) consider overall health service use rather than CMH service use specifically and/ or (ii) use area-level information which is imprecise for understanding which individuals are using these services. For example, a systematic review of UK studies found that, among people with a mental disorder, health care use was higher among females, single people, people of ethnicities other than white, and people with problems with activities of daily living [7]. However, only a subset of these studies considered community health and none considered CMH services specifically. A Canadian study estimated predictors of overall mental healthcare utilization, including CMH services (in addition to hospitalisation, primary care, and psychiatrists), and found that being female and having mental health problems such as major depressive disorder and phobia were the strongest predictors of mental healthcare use [8], and an area-level UK study found that areas with higher deprivation and unemployment, and lower proportions of people of black and other ethnic minorities, had higher overall mental health service use [9]. One study focusing specifically on CMH services found that mental health visits at community health centres have increased particularly in counties with high white populations and counties with high rates of poverty [10]. However, this study is limited due to it using area-level information and exploring only a few demographic variables. Very little is known about individual-level predictors of CMH service use specifically, partly due to the lack of population representative datasets with information on CMH service use.

This study aims to fill this gap in the literature by: (i) estimating predictors of CMH service use, including comparisons over time to explore whether demand for CMH services is changing, and (ii) discussing whether need or non-need factors are predicting use, to explore whether inequalities in access to CMH services may exist. Inequalities in healthcare utilization are commonly found in other healthcare settings (e.g. see [11]), and hence it is important to understand whether this may be occurring in CMH services also.

We conduct this study in the context of England, which is an important setting for a number of reasons. First, healthcare is free for everyone and so insurance status does not impact healthcare-seeking behaviour, meaning that any differences in use are not caused by differences in health insurance. Second, it is an example of a country where the mental healthcare system is in crisis. Lack of funding and staff shortages have been identified as key factors leading to a dysfunctional mental healthcare system that is struggling to keep up with growing demand [12]. Third, within the English National Health (NHS), CMH services play “a crucial role in delivering mental health care for adults and older adults with severe mental health needs as close to home as possible” [13]. CMH services are set to play an even larger role going forward given that a key priority for the NHS is to shift mental health care from hospitals to community settings [6, 14]. Broadly, in England CMH covers all mental health services provided outside of hospital, primary medical care settings, and talking therapies. CMH services are delivered by a variety of staff, including psychologists, psychiatrists, and psychiatric nurses. Although there is a political commitment to increasing CMH provision, there is very little evidence on where or for who these services are most needed.

We explore predictors of CMH service use with two England population representative data series: the Adult Psychiatric Morbidity Survey (APMS) and the Health Survey for England (HSE). We run logit regressions to explore how demographic, health and health needs, and area characteristics influence the likelihood that an individual used a CMH service in the past year. We examine CMH services provided in England, which have traditionally focused on clinical services such as mental health nursing, psychiatry and psychology. The ethos of these services aligns with the core principles of a recovery-based approach and providing greater accessibility to care for local populations outlined in the World Health Organisation’s definition of community mental health. However, the services we focus on are narrower in definition than those described by WHO, as we do not include broader non-clinical services or those provided in a social care (long-term care) setting due to the different arrangements for funding and provision of services provided outside of the English NHS.

Differences in CMH service use between population groups could be due to differences in need or in access (among other things), and these underlying mechanisms might have opposing effects on observed utilization rates. For example, people living in a deprived area may have higher need for CMH services. However, if inequalities in supply make it more difficult for people living in these areas to access CMH services, then they might have lower utilization rates despite their higher needs. Therefore, we consider the results in the context of existing literature on rates of mental health problems (a proxy for need), and access to healthcare services in general, to discuss what the results may imply about whether certain population groups have greater need for CMH services and/or experience inequalities in access to CMH services.

## Methods

### Data

This study uses the 2007 and 2014 waves of the Adult Psychiatric Morbidity Survey (APMS), and the 2014 wave of the Health Survey for England (HSE). These are the most recent sources of data available capturing use of CMH services among the general population in England. They were selected because they each provide individual-level response data to questions on CMH service use from a representative sample of the adult population in England. Information on CMH service use is scarce, and so we used all data sets capturing this information to maximise our ability to explore predictors of CMH service use. A benefit of using all three of these data sets (two waves from the APMS data series and one from the HSE) is that it allows us to compare results across years and between data series, and also to provide robustness for the estimates. We focus on individuals aged 18 years and over as this is the age at which people become eligible for adult mental health services within the English NHS [15].

The APMS is a survey conducted every seven years, from 1993 to 2014. Whilst a new wave of data is being collected, it is not available for use at the time of writing. The aim of the APMS survey is to provide data on the prevalence of treated and untreated psychiatric disorder in the English population aged 16+ [16, 17]. We analysed the 2007 and 2014 versions of the survey as these are the most recent versions available, and because they include information on the entire adult population (the 1993 and 2000 versions do not include participants over the age of 74). Participants for each wave of the APMS are selected using the same process, meaning comparisons can be made across years: a random, multi-stage stratified sampling method based on (i) postcode sectors to ensure an even spread across England, and (ii) random sampling of privately owned households within the postcode sectors. The 2007 version of the survey had 7,403 participants, of which 7,274 (98.3%) were aged 18 or over [16], and the 2014 version had 7,546 participants of which 7,455 (98.8%) were aged 18 or over [17].

The HSE is an annual cross-sectional survey, conducted with the aim of monitoring trends in the health of the population in England [18]. Like the APMS, the HSE is sampled first by postcode sector and then by privately owned households within each postcode sector to obtain a nationally representative sample. In addition to the core questionnaire asked every year, there are additional modules on specific topics which vary across the survey years. We analysed the 2014 wave of the survey as this contains information about CMH service use, which was asked as part of a specific module on community health. The 2014 wave of the survey had 10,080 respondents, of which 7,871 (78.1%) were aged 18 or over.

#### Outcome variable: measure of community mental health use

All three data sets elicit information on CMH service use with the same question wording, which is asked in two parts. The first part asks: “*Here is a list of community care services. Excluding any contact with professionals or team members that you have already told me about*^1^, *have you used any of these services in the last year? For example, you may have been visited at home by some of these people.*” Possible responses to this question are ‘Yes’ and ‘No’. If the respondent selects ‘Yes’, they are then asked a further question: “*Which services have you used?*” Possible responses for this question are *‘Psychiatrist’, ‘Psychologist’, ‘Community psychiatric nurse’, ‘Community learning difficulty nurse’, ‘Other nursing services’, ‘Social worker’, ‘Self-help/support group’, ‘Home help/home care worker’,* and/ or *‘Outreach worker/family support’*. Participants can respond to any number of these. We define a participant as using a CMH service in the past year if they report having used any of: ‘Psychiatrist’, ‘Psychologist’, or ‘Community psychiatric nurse’. We choose these three services to define CMH service use since they are the response options that relate specifically to CMH. These staff play a core role in delivering mental health services in England [19].

#### Independent variables: 2007 and 2014 APMS analyses

We aimed to explore what demographic, health and health needs, and area characteristics predict use of CMH services. While the question about CMH service use was the same across all three survey data sets used, information collected on other characteristics differed across the surveys/waves. When analysing the APMS data sets, we include the following demographic variables: sex (female or male), age (ten-year bands), marital status (single or not), employment status (employed or not), income (indicating if respondent is in the bottom two quintiles of household income equivalised for size and composition), education (degree or not), ethnicity (five groups), and area-level deprivation (indicating if local area of residence is in the bottom quintile of Index of Multiple Deprivation [20], where local area of residence is defined using Lower layer Super Output Areas (LSOAs) [21]). We also included variables capturing an individual’s health and health needs: whether the individual reports having activities of daily living (ADLs) that they require help with, whether they have symptoms of anxiety, depression, or phobia according to self-reported responses to questions from the Clinical Interview Schedule-Revised (CIS-R), and if they reported experiencing any aches and pains in the past month.

#### Independent variables: 2014 HSE and 2014 APMS analyses

The analysis using the HSE data used the same set of demographic variables included in the APMS analysis (sex, age, marital status, employment status, income, education, ethnicity, and deprivation), but with the addition of a variable indicating if the individual’s LSOA of residence is defined as a rural or urban area. Rurality information was not collected in the 2007 wave of the APMS, and is therefore not included in the APMS analysis which compares results across the two survey waves. However, the 2014 APMS survey does have this information and hence it was included when comparing the results from the 2014 APMS with the 2014 HSE.

Unfortunately, the AMPS and HSE do not share any identical questions covering health or health need variables. However, there are still some variables in the HSE that capture mental and physical health problems, albeit in a slightly different way. The health characteristics included in the HSE analysis are: self-reported poor mental health (classified if respondent scores higher than three in the general health questionnaire (GHQ-12)) and self-reported limiting, long-term illness.

The HSE also contains information on the government office region of residence for the respondent from which we derived seven NHS regions (London, North West, North East and Yorkshire, Midlands, East of England, South West, South East). These variables were effects coded, so the results should be interpreted as relative to the national average [22].

### Analysis

We included all respondents aged 18 years or over with complete data for the variables used in the analysis. Individuals with missing data for the GHQ-12 or income were retained using the missing indicator method, as these had the largest number of missing values. This resulted in sample sizes of 7,104 adults in the 2007 APMS data (97.7% of 18+ sample), 7,368 in the 2014 APMS data (98.8%), and 7,805 in the 2014 HSE data (99.2%).

The outcome variable of the models was a binary variable indicating if the individual reported having used any CMH service in the past year. We used logistic regression models to estimate how individual demographics, health and health needs, and area characteristics are associated with reported CMH service use.

First, we examined the predictors of CMH service use using the APMS data series. We started by estimating the models in each wave separately, and then pooled the data from the 2007 and 2014 waves. In this pooled analysis we included a dummy variable equal to 1 if the data was from the 2014 version to account for any general changes in the levels of CMH service use over time. The main advantage of the APMS data over the HSE data is the range of information on health characteristics, covering ADLs the individual needs help with, and specific physical and mental health problems.

The second set of analyses explored the predictors of CMH service use using the HSE data set. While the APMS contains more detailed information on specific mental health problems and need for help, the HSE collects data on more general health characteristics using the same question wording commonly used in other data sets. The HSE also includes information on the government office region the individual lives in, which enables exploration of possible inequalities between regions. This is an advantage over the 2014 APMS data, which does not contain any regional identifiers for individuals.

Where the APMS and HSE share the same variables, we compared the results between the 2014 HSE and the 2014 APMS to examine whether the results were similar across data series. Since the APMS and HSE both aim to obtain population representative samples, estimates using 2014 data from these two data series should provide similar results. Therefore, similar estimates between the data series would provide robustness for the estimates and evidence that the estimates are reliable predictors of CMH service use.

## Results

### Statistical analysis

#### Descriptive statistics

The descriptive statistics for the full samples, and for people who reported that they used a CMH service in the past year, are presented in Table 1. The demographics appear similar across the survey samples, with similar proportions by sex (40.26–44.18% males), age, employment status (44.91–46.91% not employed), deprivation (18.82–20.03% in the most deprived quintile), rurality (19.91–20.14% residing in a rural area). Reported CMH service use was also similar across the three data sets, ranging from 1.91% of respondents in the 2007 wave of the APMS to 2.38% in the 2014 APMS.

**Table 1:** Descriptive statistics across the three survey samples

	2007 APMS		2014 APMS		2014 HSE	
	All	Used CMH	All	Used CMH	All	Used CMH
	*N* = 7104	*N* = 136	*N* = 7368	*N* = 175	*N* = 7805	*N* = 173
Psychiatrist use (%)	0.91	47.79	1.19	50.29	1.04	46.82
Psychologist use (%)	0.58	30.15	0.80	33.71	1.02	46.24
Community psychiatric nurse use (%)	1.04	54.41	1.02	42.86	0.74	33.53
Any CMH service use (%)	1.91	–	2.38	–	2.22	–
Male	42.84	36.03	40.26	43.43	44.18	41.62
Age: Under 35	20.45	24.26	20.27	28.57*	21.59	28.90*
Age: 35 to 44	19.59	26.47**	15.91	23.43*	17.94	16.76
Age: 45 to 54	15.57	22.06**	17.45	22.29	18.81	21.39
Age: 55 to 64	17.48	16.91	16.35	13.71	15.40	17.34
Age: 65 to 74	14.03	5.88***	15.84	6.29***	15.13	8.67**
Age: 75 and over	12.87	4.41***	14.18	5.71***	11.13	6.94*
Single	43.07	62.50***	44.50	61.14***	34.91	54.34***
Not employed	45.73	67.65***	46.91	64.00***	44.91	61.85***
Low income (bottom two quintiles)	31.05	47.06***	32.37	38.29	26.42	37.57**
Missing income	19.45	21.40	21.76	26.86	19.31	19.65
Degree	19.31	17.65	24.38	23.43	25.96	20.23
Deprived (bottom quintile)	20.03	29.41*	18.82	26.29*	18.92	24.28
Ethnicity: White British	88.49	89.71	85.04	86.86	82.92	80.35
Ethnicity: White other than British	4.21	4.41	5.59	4.00	6.14	8.09
Ethnicity: Black	2.52	2.21	2.63	4.00	2.45	2.89
Ethnicity: Asian	2.94	0.74**	4.75	3.43	6.42	5.20
Ethnicity: Other	1.84	2.94	1.98	1.71	2.08	3.47
Rural	–	–	19.91	12.57**	20.14	12.14**
Self-reported poor mental health (GHQ-12 > 3)	–	–	–	–	12.81	46.24***
Missing mental health	–	–	–	–	13.43	16.76
Limiting, long-term illness	–	–	–	–	25.19	69.36***
Don’t need help with any activity	62.44	27.21	73.62	44.57	–	–
Need help with one activity	15.71	21.32	9.27	12.57	–	–
Need help with more than one activity	21.85	51.47***	17.11	42.86***	–	–
Anxiety	4.84	30.15***	6.18	33.14***	–	–
Depression	3.42	31.62***	3.73	22.29***	–	–
Phobia	2.10	24.26***	2.61	25.14***	–	–
Aches and pains	60.90	71.32**	59.42	68.00*	–	–

^***^p < 0.001; ^**^p < 0.01; ^*^p < 0.05. Statistical significance is based on t-test comparing between people using CMH services and not using CMH services

Comparing the 2014 and 2007 APMS waves, which indicates how the population has changed over time within the same data series, in 2014 we see a higher proportion of people with a degree (24.38% compared with 19.31%) and a lower proportion of people of white British ethnicity (85.04% compared with 88.49%). Comparing the health and health needs variables, there were similar rates of the three mental health problems and aches and pains, but a lower proportion of respondents reported needing help with an ADL in 2014 compared to 2007 (73.62% say they do not need help with any in 2014 compared with 62.44% in 2007).

Since the 2014 HSE and 2014 APMS both aim to be population representative samples, we would expect the samples to display similar characteristics. Comparing them, we do see that they have similar characteristics overall. However, some differences exist: we see that the 2014 HSE has a lower proportion of single people (34.91% compared with 44.50%) and of people in the lowest two income quintiles (26.42% compared with 32.37%). There is no obvious difference between the questions eliciting this information that would explain these differences.

Comparing people who reported that they have and have not used a CMH service in the past year descriptively, results across the three data sets suggest that the following characteristics were correlated with a higher probability of reporting having used a CMH service: being younger; single; not employed; on low income; living in a deprived area; and living in an urban area. The two waves of the APMS suggest that people who need help with more than one activity of daily living, have anxiety, depression, a phobia, or have experienced aches and pains in the last month are more likely to have used a CMH service in the last year. The HSE data suggests that scoring above 3 on the GHQ-12 self-reported mental health questionnaire and self-reporting having a limiting, long-term illness is associated with a higher likelihood of using a CMH service in the past year. There is no evidence that sex or education are associated with CMH service use in these raw descriptives. There is limited evidence that ethnicity is associated with CMH service use, with the only statistically significant difference found in the 2007 wave of the APMS data where people of Asian ethnicity are less likely to use a CMH service compared with people not of Asian ethnicity.

#### Model estimates

Table 2 presents the average marginal effects from logit regression models, examining the predictors of CMH use when using the APMS data. Average marginal effects can be interpreted as the average percentage point difference in the likelihood an individual used a CMH service in the past year [23].^2^ Columns 1 and 2 present the results when using the 2007 wave of the APMS, excluding and including the health variables respectively. Similarly, columns 3 and 4 present estimates using the 2014 wave, and columns 5 and 6 present these results when pooling the 2007 and 2014 waves together. Columns 7 and 8 present the pooled results but when also including interaction variables between the demographic and health variables and the year dummy variable to examine whether the relationships between these predictors and CMH use have changed over time.

**Table 2:** Predictors of CMH service use using APMS data from 2007 and 2014: average marginal effects from logistic regression models

	(1)	(2)	(3)	(4)	(5)	(6)	(7)	(8)
	2007	2007	2014	2014	Pooled	Pooled	Pooled	Pooled
Male	−0.0012	−0.0023	0.0065	$0.0072^{*}$	0.0028	0.0024	−0.0013	−0.0026
	(0.0034)	(0.0032)	(0.0036)	(0.0035)	(0.0025)	(0.0023)	(0.0038)	(0.0036)
Age: 35 to 44	0.0073	0.0008	0.0073	0.0015	$0.0073^{*}$	0.0013	0.0081	0.0009
	(0.0046)	(0.0044)	(0.0050)	(0.0048)	(0.0034)	(0.0033)	(0.0052)	(0.0050)
Age: 45 to 54	0.0072	−0.0016	0.0011	−0.0071	0.0041	−0.0040	0.0081	−0.0018
	(0.0048)	(0.0047)	(0.0051)	(0.0050)	(0.0035)	(0.0034)	(0.0054)	(0.0053)
Age: 55 to 64	−0.0067	−0.0074	$$-0.0184^{**}$$	$$-0.0218^{***}$$	$$-0.0126^{**}$$	$$-0.0147^{***}$$	−0.0075	−0.0083
	(0.0053)	(0.0050)	(0.0060)	(0.0059)	(0.0040)	(0.0038)	(0.0059)	(0.0056)
Age: 65 to 74	$$-0.0323^{***}$$	$$-0.0278^{***}$$	$$-0.0456^{***}$$	$$-0.0381^{***}$$	$$-0.0388^{***}$$	$$-0.0327^{***}$$	$$-0.0362^{***}$$	$$-0.0313^{***}$$
	(0.0078)	(0.0073)	(0.0084)	(0.0078)	(0.0058)	(0.0053)	(0.0085)	(0.0080)
Age: 75 and over	$$-0.0407^{***}$$	$$-0.0359^{***}$$	$$-0.0509^{***}$$	$$-0.0453^{***}$$	$$-0.0457^{***}$$	$$-0.0405^{***}$$	$$-0.0456^{***}$$	$$-0.0405^{***}$$
	(0.0089)	(0.0083)	(0.0088)	(0.0083)	(0.0062)	(0.0059)	(0.0096)	(0.0091)
Degree	0.0035	0.0057	0.0029	0.0061	0.0032	$0.0059^{*}$	0.0039	0.0064
	(0.0045)	(0.0043)	(0.0044)	(0.0043)	(0.0031)	(0.0030)	(0.0050)	(0.0048)
Ethnicity: White other than British	−0.0027	−0.0036	−0.0141	−0.0074	−0.0084	−0.0052	−0.0030	−0.0041
	(0.0079)	(0.0078)	(0.0090)	(0.0083)	(0.0060)	(0.0056)	(0.0088)	(0.0088)
Ethnicity: Asian	−0.0310	−0.0308	$$-0.0228^{*}$$	−0.0171	$$-0.0228^{**}$$	$$-0.0187^{*}$$	−0.0347	−0.0347
	(0.0186)	(0.0174)	(0.0098)	(0.0091)	(0.0081)	(0.0075)	(0.0207)	(0.0195)
Ethnicity: Black	−0.0120	−0.0166	−0.0023	0.0025	−0.0063	−0.0064	−0.0134	−0.0187
	(0.0110)	(0.0106)	(0.0092)	(0.0087)	(0.0068)	(0.0066)	(0.0123)	(0.0119)
Ethnicity: Other	0.0022	−0.0006	−0.0151	−0.0077	−0.0059	−0.0034	0.0025	−0.0007
	(0.0097)	(0.0095)	(0.0135)	(0.0125)	(0.0081)	(0.0077)	(0.0108)	(0.0107)
Low income	0.0049	−0.0008	−0.0025	−0.0048	0.0012	−0.0028	0.0055	−0.0009
	(0.0043)	(0.0041)	(0.0048)	(0.0046)	(0.0032)	(0.0031)	(0.0048)	(0.0047)
Missing income	0.0049	0.0030	0.0061	0.0050	0.0058	0.0039	0.0054	0.0034
	(0.0048)	(0.0045)	(0.0049)	(0.0047)	(0.0034)	(0.0033)	(0.0054)	(0.0051)
Deprived	0.0006	−0.0025	0.0014	−0.0042	0.0012	−0.0030	0.0007	−0.0028
	(0.0038)	(0.0037)	(0.0042)	(0.0042)	(0.0028)	(0.0028)	(0.0042)	(0.0041)
Not employed	$$0.0272^{***}$$	$$0.0151^{***}$$	$$0.0341^{***}$$	$$0.0179^{***}$$	$$0.0310^{***}$$	$$0.0168^{***}$$	$$0.0304^{***}$$	$$0.0170^{***}$$
	(0.0045)	(0.0041)	(0.0047)	(0.0045)	(0.0033)	(0.0030)	(0.0048)	(0.0045)
Single	$$0.0136^{***}$$	$$0.0074^{*}$$	$$0.0135^{***}$$	0.0054	$$0.0135^{***}$$	$$0.0064^{**}$$	$$0.0152^{***}$$	$$0.0084^{*}$$
	(0.0036)	(0.0034)	(0.0039)	(0.0037)	(0.0027)	(0.0025)	(0.0040)	(0.0038)
Need help with one activity		$$0.0198^{***}$$		$$0.0176^{**}$$		$$0.0185^{***}$$		$$0.0223^{***}$$
		(0.0047)		(0.0056)		(0.0035)		(0.0051)
Need help with more than one activity		$$0.0212^{***}$$		$$0.0245^{***}$$		$$0.0223^{***}$$		$$0.0239^{***}$$
		(0.0047)		(0.0048)		(0.0033)		(0.0051)
Anxiety		$$0.0157^{***}$$		$$0.0219^{***}$$		$$0.0182^{***}$$		$$0.0177^{***}$$
		(0.0042)		(0.0046)		(0.0031)		(0.0047)
Depression		$$0.0194^{***}$$		0.0025		$$0.0118^{***}$$		$$0.0219^{***}$$
		(0.0044)		(0.0055)		(0.0035)		(0.0049)
Phobia		$$0.0183^{***}$$		$$0.0283^{***}$$		$$0.0224^{***}$$		$$0.0206^{***}$$
		(0.0047)		(0.0054)		(0.0036)		(0.0052)
Aches and pains		−0.0054		−0.0006		−0.0026		−0.0061
		(0.0037)		(0.0039)		(0.0027)		(0.0042)
Year = 2014					$0.0055^{*}$	$0.0059^{*}$	0.0114	0.0095
					(0.0024)	(0.0024)	(0.0079)	(0.0077)
**Interactions with Year = 2014**								
Male							0.0072	0.0091
							(0.0050)	(0.0048)
Age: 35 to 44							−0.0015	0.0004
							(0.0069)	(0.0066)
Age: 45 to 54							−0.0071	−0.0047
							(0.0071)	(0.0070)
Age: 55 to 64							−0.0092	−0.0114
							(0.0079)	(0.0076)
Age: 65 to 74							−0.0052	−0.0031
							(0.0110)	(0.0104)
Age: 75 and over							−0.0006	−0.0004
							(0.0119)	(0.0114)
Degree							−0.0013	−0.0009
							(0.0064)	(0.0062)
Ethnicity: White other than British							−0.0098	−0.0026
							(0.0120)	(0.0115)
Ethnicity: Asian							0.0140	0.0192
							(0.0225)	(0.0211)
Ethnicity: black							0.0113	0.0209
							(0.0148)	(0.0143)
Ethnicity: other							−0.0162	−0.0062
							(0.0163)	(0.0156)
Low income							−0.0078	−0.0034
							(0.0065)	(0.0062)
Missing income							0.0001	0.0010
							(0.0070)	(0.0066)
Deprived							0.0005	−0.0010
							(0.0057)	(0.0056)
Not employed							0.0005	−0.0008
							(0.0059)	(0.0059)
Single							−0.0030	−0.0035
							(0.0052)	(0.0050)
Need help with one activity								−0.0064
								(0.0070)
Need help with more than one activity								−0.0018
								(0.0065)
Anxiety								0.0020
								(0.0061)
Depression								$$-0.0196^{**}$$
								(0.0070)
Phobia								0.0049
								(0.0071)
Aches and pains								0.0056
								(0.0054)
BIC	1364.2497	1267.3021	1656.2339	1559.8763	2900.8700	2671.7122	3044.0562	2859.0708
Observations	7104	7104	7368	7368	14472	14472	14472	14472

^***^p < 0.001; ^**^p < 0.01; ^*^p < 0.05

The results are mostly similar between the 2007, 2014, and pooled data sets, showing consistent relationships over time and across survey samples. We find that people who are: single; younger; not employed; who need help with an ADL; and have a mental health problem are more likely to use a CMH service. There is also evidence that people of Asian ethnicity are less likely to use a CMH service compared with people of White British ethnicity (although the marginal effect is not statistically significant in the 2007 models, the magnitude of effect is in fact largest for these models, suggesting that the lack of significance may be due to lower statistical power rather than a structural change in CMH use for this population group over time).

Comparing the 2007 and 2014 waves from the APMS, we find evidence of an increase in CMH service use over time. When including the full set of variables, the probability that an individual reports having used CMH services was 0.59% points higher in 2014 compared to 2007 (column 6), representing a 30.9% increase over this seven year period. This may signal growing demand for CMH services, possibly caused by rising rates of mental health problems (beyond the measures of mental health included in our models) or a reduction in stigma surrounding mental health. In terms of predictors of CMH service use, the similarity of the results for 2007 in columns 1 and 2 with the 2014 results in columns 3 and 4 suggest that the characteristics that predict CMH service use have remained fairly stable over time. The only notable difference is that the 2014 wave suggests that males are more likely to use a CMH service, where the 2007 data set does not. This might imply that between 2007 and 2014 males became relatively more likely to use a CMH service. However, as discussed in more detail below, there is no evidence that males are more likely to use a CMH service when using the 2014 HSE data and so this result should be interpreted with caution. The average marginal effects for the interaction terms in columns 7 and 8 provide further evidence that predictors of CMH service use have remained relatively stable over time, with only one of the interaction terms statistically significant at the 5% level. The depression and year interaction suggests that depression is a greater predictor of CMH service use in the 2007 wave than the 2014 wave, consistent with a lower marginal effect for depression in column 4 (0.0025) compared with column 2 (0.0195).

Table 3 presents the average marginal effects from logit regression models using the 2014 HSE data, and one model using the 2014 APMS data for comparison. Columns 1 and 2 present the model results when using the 2014 APMS and the 2014 HSE including demographic variables only. Columns 3 to 5 add the mental and physical health variables for the HSE data, and column 6 includes the NHS region of residence for the individual.

**Table 3:** Predictors of CMH service use using HSE and APMS data from 2014: average marginal effects from logistic regression models

	2014 APMS	2014 HSE				
	(1)	(2)	(3)	(4)	(5)	(6)
Male	0.0067	0.0010	0.0016	0.0015	0.0020	0.0022
	(0.0036)	(0.0034)	(0.0033)	(0.0033)	(0.0033)	(0.0033)
Age: 35 to 44	0.0074	−0.0002	−0.0026	−0.0076	−0.0076	−0.0076
	(0.0050)	(0.0052)	(0.0051)	(0.0051)	(0.0051)	(0.0050)
Age: 45 to 54	0.0015	0.0041	0.0009	−0.0063	−0.0064	−0.0059
	(0.0051)	(0.0049)	(0.0048)	(0.0048)	(0.0048)	(0.0048)
Age: 55 to 64	$$-0.0179^{**}$$	−0.0029	−0.0040	$$-0.0142^{**}$$	$$-0.0130^{*}$$	$$-0.0133^{*}$$
	(0.0060)	(0.0052)	(0.0051)	(0.0053)	(0.0052)	(0.0052)
Age: 65 to 74	$$-0.0448^{***}$$	$$-0.0257^{***}$$	$$-0.0214^{**}$$	$$-0.0338^{***}$$	$$-0.0290^{***}$$	$$-0.0295^{***}$$
	(0.0084)	(0.0069)	(0.0067)	(0.0069)	(0.0067)	(0.0067)
Age: 75 and over	$$-0.0505^{***}$$	$$-0.0291^{***}$$	$$-0.0256^{***}$$	$$-0.0417^{***}$$	$$-0.0360^{***}$$	$$-0.0353^{***}$$
	(0.0088)	(0.0075)	(0.0073)	(0.0076)	(0.0075)	(0.0074)
Degree	0.0030	−0.0026	−0.0030	0.0001	−0.0002	−0.0008
	(0.0044)	(0.0044)	(0.0044)	(0.0044)	(0.0043)	(0.0043)
Ethnicity: White other than British	−0.0149	0.0050	0.0053	0.0073	0.0072	0.0044
	(0.0090)	(0.0063)	(0.0062)	(0.0062)	(0.0061)	(0.0063)
Ethnicity: Asian	$-0.0239^{*}$	−0.0090	−0.0081	−0.0054	−0.0057	−0.0074
	(0.0098)	(0.0077)	(0.0076)	(0.0075)	(0.0074)	(0.0075)
Ethnicity: Black	−0.0033	−0.0036	−0.0027	0.0046	0.0043	0.0011
	(0.0092)	(0.0101)	(0.0100)	(0.0099)	(0.0099)	(0.0100)
Ethnicity: Other	−0.0160	0.0039	0.0043	0.0085	0.0091	0.0055
	(0.0135)	(0.0093)	(0.0092)	(0.0092)	(0.0092)	(0.0094)
Low income	−0.0027	0.0037	−0.0001	−0.0000	−0.0023	−0.0014
	(0.0048)	(0.0041)	(0.0041)	(0.0041)	(0.0041)	(0.0041)
Missing income	0.0061	−0.0001	−0.0023	−0.0022	−0.0034	−0.0031
	(0.0049)	(0.0047)	(0.0047)	(0.0046)	(0.0046)	(0.0046)
Deprived	−0.0001	−0.0020	−0.0039	−0.0050	−0.0056	−0.0050
	(0.0042)	(0.0041)	(0.0041)	(0.0041)	(0.0040)	(0.0042)
Not employed	$$0.0342^{***}$$	$$0.0228^{***}$$	$$0.0173^{***}$$	$$0.0120^{**}$$	$$0.0095^{*}$$	$$0.0092^{*}$$
	(0.0047)	(0.0042)	(0.0041)	(0.0041)	(0.0041)	(0.0041)
Single	$$0.0132^{***}$$	$$0.0147^{***}$$	$$0.0111^{**}$$	$$0.0113^{**}$$	$$0.0092^{**}$$	$$0.0086^{*}$$
	(0.0039)	(0.0036)	(0.0035)	(0.0035)	(0.0034)	(0.0034)
Rural	−0.0095	$-0.0109^{*}$	$-0.0106^{*}$	$-0.0104^{*}$	−0.0098	−0.0078
	(0.0054)	(0.0052)	(0.0051)	(0.0051)	(0.0050)	(0.0051)
Self-reported poor mental health			$$0.0375^{***}$$		$$0.0257^{***}$$	$$0.0259^{***}$$
			(0.0044)		(0.0041)	(0.0041)
Missing mental health			$$0.0183^{***}$$		$$0.0125^{*}$$	$$0.0117^{*}$$
			(0.0050)		(0.0049)	(0.0048)
Limiting, long-term illness				$$0.0435^{***}$$	$$0.0359^{***}$$	$$0.0355^{***}$$
				(0.0046)	(0.0045)	(0.0044)
London						$0.0089^{*}$
						(0.0043)
East of England						−0.0068
						(0.0055)
Midlands						0.0028
						(0.0036)
North East and Yorkshire						0.0035
						(0.0036)
North West						−0.0098
						(0.0051)
South East						$0.0075^{*}$
						(0.0036)
South West						−0.0061
						(0.0057)
BIC	1661.7268	1728.0047	1647.6039	1594.0363	1566.9412	1606.5158
Observations	7368	7805	7805	7805	7805	7805

^***^p < 0.001; ^**^p < 0.01; ^*^p < 0.05

Consistent with the results from the APMS data, the results from the HSE data provide evidence that younger people, people who are not employed, and single people are more likely to use a CMH service (the effect of age is stronger in the APMS data, but a significant effect exists in both data series). The HSE data also suggests that people living in a rural area are less likely to use a CMH service. Although this is not statistically significant when using the APMS data, it is similar in magnitude to that in the HSE data.

Columns 3 to 5 suggest that scoring above 3 on the GHQ-12 and/ or having a limiting, long-term illness predict a higher likelihood of using a CMH service. The results in column 6 suggest that people living in the South East or London NHS regions are more likely to use a CMH service, and people living in the North West are less likely to use a CMH service, compared with the national average (although the result for the North West is not statistically significant at the 5% level, it is largest magnitude of marginal effect among the seven NHS regions and hence is a notable finding).

Comparing columns 1 and 2, we see that the results are quite similar between the data series, providing evidence of robust results across the two surveys. One notable difference is that, as when comparing with the 2007 APMS results, the marginal effect of male is larger for the 2014 sample of respondents from the APMS compared with the 2014 HSE sample, again suggesting that the sex differences detected in the 2014 APMS be interpreted with caution. The other key difference between the two data sets is the magnitude of marginal effect for people of Asian ethnicity, which is smaller and not statistically significant in the HSE data.

Note that across Tables 2 and 3, the average marginal effects for older people, single people, and people who are not employed typically decrease in magnitude when additional health variables are added to the models. This suggests that part of the reason why people of these demographics are more or less likely to use CMH services is due to differences in need for CMH services, which are subsequently partially captured by the health variables in the later models. However, note that the average marginal effects generally remain statistically significant, suggesting that there are differences in CMH service use beyond these measured health characteristics.

### Need for CMH services and inequalities in access

In this section of the paper, we consider the results of each demographic characteristic in the context of existing literature to discuss what the results may imply about differences in need for, and access to, CMH services between population groups. The logic here is that if a demographic characteristic is typically associated with higher rates of mental health problems, we would expect to see a higher probability of CMH service use for this population group. If this is the case, then we contend that the result is driven largely by greater need. However, if we do not detect a higher likelihood of CMH service use amongst population groups known to experience higher prevalence of mental health problems, we suggest this as evidence for an inequality in access, particularly in cases when there are examples of inequalities in access to other mental healthcare services.

We found evidence that younger people, single people, and people who are not employed are more likely to use a CMH service. These relationships are apparent both in the descriptive statistics when not controlling for other characteristics, and in the model results when controlling for demographics, health characteristics, and regions. This is consistent with existing literature which finds that younger, single, and not employed people are typically more likely to have mental health problems and more likely to use other types of mental healthcare services (e.g. see [24–27] ). Together this suggests that our results are driven by greater need for CMH services among these population groups. Note that for each of these characteristics, the average marginal effects are generally smaller in the models that include health characteristics (although mostly remain statistically significant). These health characteristics are likely to partly capture need for CMH services, and hence this provides further evidence that the reason younger, single, and not employed people are more likely to use a CMH service is at least partly due to these demographics having greater need for these services.

Overall, we found limited evidence for any differences in CMH service use by biological sex. There are no statistically significant differences in the descriptive statistics, and we detect evidence that males are more likely to use a CMH service when controlling for other demographic and health characteristics in the 2014 APMS data only. Existing literature suggests that females are typically more likely than males to experience mental health problems such as depression and anxiety [24, 28], and that they are more likely to use wider mental healthcare [25, 29]. We therefore would expect females to have higher CMH service use, and hence our null findings may indicate barriers in access to CMH services for females. However, given the lack of consistent evidence across the statistical analyses, it is difficult to conclude any differences in need for, or access to, CMH services based on sex.

Existing literature has found that people with lower income, lower education, and who live in deprived areas are typically more likely to have mental health problems [24, 30] but have greater difficulty accessing mental healthcare services [25, 31, 32] and health care in general [33, 34]. This indicates potentially greater need for CMH services among these populations, but possible inequalities in access. Therefore, if our findings indicated lower CMH service use among people with low income, low education, or who live in more deprived areas it would signal likely inequalities in access. However, regarding education, there is little evidence in either the descriptive statistics or the models that it predicts CMH service use. Similarly for income and deprivation, although the descriptive statistics indicate that people with lower income and who live in more deprived areas are more likely to use CMH services (which could be due to greater need), the results from the models when controlling for other characteristics found no such evidence that these are predictors of CMH service use. This suggests that the results from the descriptive statistics are more driven by other characteristics correlated with low income and deprivation, such as not being employed. These results hence provide no significant evidence that income, education, and deprivation are sources of inequality in access to CMH services.

Ethnic minorities are another example of a demographic group which existing literature predicts is associated with worse mental health [24, 35] but lower use of mental healthcare, particularly for people of Asian ethnicity [27, 36]. We found some evidence that people of Asian ethnicity have lower use of CMH services, both in the descriptive statistics and in the models when controlling for other demographic and health variables. Given that Asian ethnicity is typically associated with worse mental health than the general population, and possible inequalities in mental healthcare use generally, this indicates a potential inequality in access to CMH services.

The final demographic characteristic we explored is rurality. We found evidence, both in the descriptive statistics and the models, that people living in rural areas have a lower likelihood of using a CMH service. In the model, the marginal effect barely changed when including mental and physical health variables, suggesting that this result is not driven by lower need and may instead be due to an inequality in access/ supply. This potential inequality in access is often seen in other health care settings [37], including general community health services [38]. Further, when comparing regions, we found that people who live in London and the South East are more likely to use CMH services, and people living the North West are less likely to use CMH services, compared with the national average. This suggests that the North West of England may be experiencing inequalities in access to CMH services relative to other regions.

## Discussion

There is a global push to increase provision of mental healthcare in community settings. However, little is known about where or for who these services are most needed. This study aimed to explore predictors of CMH service use to better understand: (i) predictors of demand for these services, and (ii) possible sources of inequality in supply and access to these services. We did this by using three separate data sets with information about past-year use of CMH services, and comparing the results with existing literature to postulate whether differences in use between populations groups is due to differences in need for services and/ or inequalities in access to CMH services.

There was consistent evidence that being younger, single, and not employed is associated with a higher likelihood of CMH service use. There was some evidence that people of Asian ethnicity and people living in rural areas and/ or the North West NHS region are less likely to have used a CMH service in the past year. There was limited evidence that sex, education, income, or deprivation predict CMH service use. Based on existing literature about the association between the demographic variables and both prevalence of mental health problems and access to healthcare, we hypothesize that our findings are evidence that being younger, single, and not in employment are predictors of greater need for CMH services. We also find evidence that some possible inequalities exist. In particular, we find evidence that people of Asian ethnicity and people living in rural areas and/ or in the North West have lower access to CMH services, whereas people living in the South East or London have greater access. Finally, we found evidence that CMH service use was higher in 2014 compared with 2007, which could indicate growing demand for CMH services and/ or improved access to CMH care.

One limitation of this study is the lack of recent available data. The most recent population representative data sets containing information about CMH service use are from 2014. The CMH service landscape may have changed since then, which could influence the results. However, note that we found similar predictors in our 2007 and 2014 samples, which suggest that these predictors are relatively stable over time and hence are likely to still be relevant now. We can apply our findings to recent estimates of population characteristics to discuss how recent demographic shifts may have impacted overall levels of CMH service use since this was last observed in the 2014 survey. Focusing on the three characteristics we identified as significantly predicting higher need for CMH services (single, not employed, and age below 65), we consult population data from the Office for National Statistics (ONS) to examine how these three drivers of CMH service use have changed between 2014 and 2022 (2022 chosen as this is the most recent year with ONS estimates for marital status). The proportion of adults who are single has increased from 48.5% to 50.6% [39], which would suggest an increase in demand for CMH. However, our estimates suggest that the increase in the employment rate amongst the working age population over the same period may have eased demand for CMH, as employment rose from 72.9% to 75.1% [40]. The percentage of the population aged below 65 has decreased marginally during this period from 63.7% to 62.9% [41], which may reduce rates of CMH use per head of population. Taken together this suggests that demographic shifts in the drivers of CMH use since the most recent survey was undertaken are likely to have had multiple divergent effects on overall demand for CMH services. Note that this interpretation applies if the relationships between the predictors and CMH service use remain stable over time. We find evidence that the relationships are relatively stable between 2007 and 2014. Future research should examine whether this also holds between 2014 and more recent data when that becomes available. A second limitation is that it is hard to disentangle the potentially opposing effects of differences in need for, and access to, CMH services. For example, null results such as that for deprivation does not mean there is no inequality in access. It is possible that people living in deprived areas have lower access but greater need, in which case these two effects could cancel each other out and provide a null overall result. Further, lower use of CMH services may not just be due to lower need or supply, but may instead be due to other factors such as differences in preferences or stigma surrounding mental health treatment seeking. A third limitation is that the data used self-reported information on CMH service use, which may be subject to sources of inaccurate data such as recall-bias or non-disclosure problems [42, 43]. However, self-reported measures of healthcare utilization are typically fairly accurate [44]. Further, this study gathered information on ‘any CMH service use’ rather than the specific number of uses within the year, which should yield more accurate responses.

## Conclusions

This study provides valuable information about predictors of CMH service use, a mental health service for which there is currently little information. The World Health Organisation has emphasised the importance of up-scaling community mental health [4], and in countries such as England there is a long-term focus to increase CMH services to shift some of the burden of rising rates of mental health problems away from hospitals [6]. The findings in this study provide valuable information to identify demographics with greater need for CMH services, and potential inequalities in access to CMH services, that can help to highlight targets for increases in CMH services. The findings suggest allocating more resources to areas with higher rates of younger, single, and not employed people, as these are groups with greater demand for services, and rural areas, the North West, and areas with higher rates of people of Asian ethnicity, given evidence of barriers to access for these groups. This can help to reduce the strain on mental health services and improve mental health outcomes. Future research should examine whether the identified relationships are the same following the COVID pandemic remain stable over time when more up-to-date data on CMH service use becomes available.

## Data Availability

All data used in this study is available through the UK data service. Registration for the UK data service is required for all data sets. Special License access is required for the 2014 wave of the APMS.

## Footnotes

1 Health services that the respondent is asked about prior to this question include hospitals, GP, and day activity services.

2 Since logit regressions are non-linear models, the marginal effects differ depending on the characteristics of the individual. Here, average marginal effects are presented, which are the marginal effects for each individual averaged across the entire sample.
